# A model for the facilitation of effective management of aggression experienced by Psychiatric Nurses from patients in a psychiatric institution

**DOI:** 10.4102/curationis.v39i1.1676

**Published:** 2016-11-30

**Authors:** Emmanuel Bimenyimana, Marie Poggenpoel, Annie Temane, Chris Myburgh

**Affiliations:** 1 Department of Nursing Science, University of Johannesburg, South Africa

## Abstract

**Background:**

‘The time I was hit nobody helped me. They (psychiatric nurses) just said: “you do not have to worry, you are not bleeding … in time you will see more”’. The core of the nursing profession is caring for those in need. However, when the psychiatric nurse (PN) is overwhelmed by aggression from the person cared for, the ideal of rendering quality patient care is compromised. There has to be a way to assist the PNs to manage aggression effectively in order to render quality patient care and improve PNs mental health.

**Objectives:**

The purpose of this article is to describe the process that was followed in developing, describing and evaluating a model that could be used as a framework of reference to facilitate the effective management of aggression as an integral part of the mental health of PNs.

**Methods:**

A theory-generative, qualitative, exploratory, descriptive and contextual study design was used to develop the model. The central concept was derived from the Masters’ research ‘The lived experience of aggression and violence by PNs in a Gauteng psychiatric institution’. The process entailed the identification of the central concept, the definition of the central concept and other essential criteria and the classification of the central and related concepts. The conceptual framework was then described and evaluated.

**Results:**

The central concept was identified and the ‘facilitation of effective self-management of aggression’ was described and evaluated.

**Conclusion:**

The conceptual framework may be able to assist PNs in managing aggression effectively, taking control of workplace environment.

## Introduction

Aggression in psychiatric institutions is a worldwide phenomenon (Drach-Zahavy *et al*. 2012:43–45). The research has also found that high rates of aggression impact on the quality of care provided to the patient (Franz *et al*. 2010:1). In managing aggression, researchers have prioritised the identification of factors that increase the likelihood that a patient will act in an aggressive way (Drach-Zahavy *et al*. 2012); however, Van Wijk, Taut and Julie (2014) found that appropriate training in preventive standard measures provided to psychiatric nurses (PNs) could assist in addressing the challenge of aggression by psychiatric patients. A better management of workplace aggression must not view it as an individual problem but rather as a structural, strategic problem rooted in social, economic, organisational and cultural factors (Van Wijk *et al*. 2014). The first step would be to secure the environment and make PNs feel safe and have confidence to manage aggression; it should also be kept in mind that, as no single factor can stand alone as the cause of aggression (Agency for Healthcare Research and Quality 2015; Daffern *et al*. 2010:369; Laiho *et al*. 2014), the same single approach cannot yield a lasting solution.

In the context of South Africa, there is no comprehensive departmental guideline on how aggression is dealt with in psychiatric institutions. The lack of formal baseline data on the management of aggression (Bock 2011:21) has resulted in PNs managing aggression in the best way they see fit.

### Problem statement

Given the type of patients the PNs care for, it is inevitable that they meet with aggressive incidents (James, Isa & Oud 2011:130) especially in the case of a psychiatric institution where the main criterion for admission is for the patient to be disruptive and unmanageable in general hospitals and clinics. A patient’s aggressive behaviour leads to adverse consequences for PNs (Drach-Zahary *et al*. 2012; Van Wijk *et al*. 2014) including fear, anger, frustration and abuse of substances (Bimenyimana *et al*. 2009:8–9). Despite the negative experiences of PNs, very little has been done to put in place a mechanism that can assist PNs to deal with aggression effectively.

#### Research purpose

The purpose of the research study was to develop, describe and evaluate a model that can be used as a framework of reference for the advanced Psychiatric Nurse Practitioner (PNP) to assist PNs in the effective management of aggression from the patients.

#### Research objectives

The research study objectives were the following:

- to derive from the researcher’s Masters’ research central concepts to be utilised in the model, to define and classify them;
- to describe relationships between these concepts;
- to develop, describe and evaluate a model as a framework of reference for the advanced PN practitioner to facilitate effective management of aggression experienced by PNs working in a psychiatric institution.

#### Definition of key concepts

After key concepts analysis, the central concept was identified as the ‘effective self-management of aggression’. The key concepts model, facilitation, effective, self-management and aggression were defined.

#### Model

Chinn and Kramer (2011:157) define a model as a symbolic representation of an empirical experience in the form of words, pictorial or graphic diagrams, mathematical notations or physical material. In this research study, a model structure was developed as a framework of reference and illustrated what the advanced PN practitioner was to do in facilitating the effective self-management of aggression experienced by PNs.

#### Facilitation

The concept of facilitation is understood as a dynamic interactive process for the promotion of mental health through the creation of a positive environment and mobilisation of resources as well as the identification and bridging of obstacle in the promotion of mental health (University of Johannesburg 2012:7). In this research study, facilitation was defined as a dynamic interactive process through which the advanced PNP guides and provides assistance to the PNs in making easier the effective self-management of aggression.

#### Effective

According to Wehmeier, McIntosh and Turnbull (2005:469), effective means producing the result that is wanted or intended. In this research study, effective referred to the use of operative alternative means by PNs aimed at producing the intended result, which is their self-management of aggression.

#### Self-management

One of The Oxford Advanced Learner’s Dictionary’s (Hornby 2010:902) definitions of ‘management’ is, that it is the ‘act or skill of dealing with people or situations in a successful way’. The researcher was convinced that to manage other people, one should start with self or self-management. In this research study, ‘self-management’ was defined for the PNs as the ability to regulate their emotions so that they can take responsibility for, and can implement actions for, needed change resulting in taking control of their lives and self-managing aggression effectively.

#### Aggression

Franz *et al*. (2010:1), Irwin (2006:309), and Ketelsen *et al*. (2007:92) define aggression as a behaviour characterised by anger; hostile thoughts, words and actions towards others that manifests in speech, tone of voice, body language, outward expression of anger or rage; and threatening, actual or physical. In this research study, aggression meant ‘any act or omission that resulted in physical, psychological, emotional, or spiritual discomfort’. It entails verbal, non-verbal or physical expressions such as swearing, hostile looks, threats, indecency, inappropriate touch, consciously withholding information regarding one’s health, intentional non-cooperation, embarrassing someone and ultimately using physical force. The extreme expression of aggression that results in physical harm, such as in beating or injuring someone, was described, in the research study, as ‘violence’.

#### Psychiatric institution

A psychiatric institution is defined as a mental healthcare facility where care, treatment and rehabilitation are provided in accordance with the rules and regulations of the Department of Health as stipulated in the *Mental Health Care Amendment Act 12 of 2014* (Government Gazette, no 37693). In this research study, a psychiatric institution referred to is a third-level referral hospital where adults and adolescents, male or female, general or forensic patients are referred for care, treatment and rehabilitation because of their being unmanageable in other healthcare facilities.

It is also important to note that in the concept of advanced PNP, a PN is referred to as a person with a post-basic qualification and having both theoretical and clinical competencies in the management of aggressive incidents.

## Research design and method

### Design

A qualitative, exploratory, descriptive, contextual and theory-generative design was utilised in the model development and implementation (Burns & Grove 2009:51–64; 507–547; Chinn & Kramer 2011:156–182; Grove, Gray & Burns 2015). The qualitative research design allows an attempt to make sense of, or interpret, phenomena in terms of the meanings people bring to them (Denzin & Lincoln 2008:4); making the world of an individual visible to the rest of other people (LoBiondo-Wood & Haber 2010:86). Descriptive design refers to an empirically based assessment of what evaluation looks like, under different conditions, and what kinds of consequences result from various approaches to evaluation (Smith & Brandon 2008:114). The contextual dimension of the research refers to an analysis of social and historical processes, and the worth or validity of the project depends on how thoroughly and defensibly or correctly this has been done (Denzin & Lincoln 2008:461). A theory is a set of interrelated concepts, definitions and propositions that present a systematic view of phenomena for the purpose of explaining and making predictions about those phenomena (LoBiondo-Wood & Haber 2010:587). Theory development provides a way to identify and express key ideas about the essence of practice (Walker & Avant 2011:3).

A postmodern constructivist philosophy of science was utilised throughout the study. Postmodernism is a theory and the basic concept is that knowledge claims must be set within the conditions of the world as it is today and in the multiple perspectives of class, race, gender and other group affiliations. Social constructivism is an approach where individuals seek the meaning of the world in which they live and work. The meaning that individuals develop is subjective and derives from their experiences of certain objects or things. These meanings are not simply imprinted on individuals but are formed through historical and cultural norms that operate in individuals’ lives (Creswell 2014:8). This means that how one divides up the world in order to understand it is the result of historical, social and political processes (Green & Thorogood 2009:15). Central to social constructivism is the attempt to understand scientific activity and scientific knowledge in social terms, as arising from interactions between various participating agents in the social domain (Quale 2008:59).

### Method

The four steps of Chinn and Kramer (2011:163–182) for theory development were used. These four steps are concept analysis, relationship statements, description of the model and the evaluation of the model.

#### Step one: Concept analysis

Concept analysis took place in two phases. In phase one, the central concept was identified, and in phase two, the identified concept were defined and classified.

*Phase one:* Identification of the central concept

The central concepts were identified through the field works that were conducted.

*Phase two:* Definition and classification of concepts

Concepts were defined using hard copies, online dictionaries and subject-specific literature. Concepts were given meaning, or the meaning they represented was expressed, and linguist representations of the concepts were expressed in empiric reality (Chinn & Kramer 2011:165).

The survey list of Dickoff, James and Wiedenbach (1968:434–450) was utilised to classify the following concepts and comprised the agent, the recipient, the procedure, the dynamic, the context and the outcome as discussed below.

#### Step two: Describing relationships between concepts

The identified and defined concepts in step one were put into interrelated statements (Chinn & Kramer 2008:246–248, 2011:180).

#### Step three: Description of the model

The description of the model was based on the six components suggested by Chinn and Kramer (2008:235–248). These components are purpose, concepts, definitions, relationships, structure and assumptions (Chinn & Kramer 1995:106–123). The model’s structure and process were described. After providing a description of the model on structural and process aspects, the guidelines for the implementation of the model were provided.

#### Step four: Evaluation of the model

The evaluation of the model used the Chinn and Kramer (2008:235–248) evaluation criteria of clarity, simplicity, generality, accessibility and importance of the model. The model was evaluated throughout by three experts: two professors and one Ph.D. holder, who are the supervisor and co-supervisors. The model was also evaluated during the annual research forum organised by the University of Johannesburg. On all these occasions, the model was found to comply with the above criteria for model development.

## Ethical considerations

The ethical principles that guided this research study are stipulated by the Medical Research Council of South Africa (MRC 2003:2–6) as published on their website (www.mrc.co.za) and the United Nations Educational, Scientific and Cultural Organisation (UNESCO 2006:23–31). These principles are autonomy or respect for persons, beneficence, non-maleficence and justice. The respect for the person underlies the requirement that human participants decide and give their free, voluntary and informed consent to participate in research (Dhai & McQuoid-Mason 2011:14). The participants in the study were PNs who are able to make a sound and independent judgement about their actions. An invitational letter was sent to the participants setting out the research purpose, content and process and the participants freely signed an informed consent (UNESCO 2006:25) after they had been given satisfactory information. Permissions from ethical committees of both the University of Johannesburg (AEC77/02-2010) and Wits University (M110114) were obtained; permission was also obtained from the higher degree committee of the Faculty of Health Sciences (HDC69/02-2010) as well as the Chief Executive Officer of the hospital. Non-maleficence: the researcher ensured that no harm occurs to the participants (Polit & Beck 2008:170). A contingency measure was in place in case any participant would need it though no harm had been foreseen. Beneficence represents the opposite of harm and refers to any favourable outcome of the research to the society or to the individual (MRC 2003:2–6). The study intended to sustain the well-being of the participants (Tolich & Sieber 2013:42). Justice was served by treating all the participants equally, respecting them and acknowledging their right to decide what is good for them (Dhai & McQuoid-Mason 2011:15).

## Measures to ensure trustworthiness

Guba’s model of trustworthiness criteria (Lincoln & Guba 1985:301–331) was used to ensure the research study’s trustworthiness. The criteria of credibility, transferability, dependability and confirmability were followed.

De Vos *et al*. (2011:419–420) state that the goal of credibility is to demonstrate that the research was conducted in such a manner that there is a match between research participants’ views and the researcher’s construction and representation of them. The researcher ensured credibility by using various methods in data collection (triangulation) including interviews, field notes, naive sketch writing and observation as well as maintaining a reflexive journal in which all his experiences and observations during the model process were noted. The model structure was presented in a doctoral seminar and recommended adjustments were made. The sampling method used was a purposive sampling, and a dense description of data collection and analysis as well as the participants’ profile description were provided in detail together with verbatim quotes to enable the reader to determine whether the results are likely transferable (Mateo & Kirchhoff 2009:151). Dependability was enhanced by the researcher’s description of the steps taken and supported them with literature review providing a clear understanding and describing every step before it was taken and the whole process was overseen by both the researcher and the research study supervisor and co-supervisors. Confirmability entailed being objective (De Vos *et al*. 2011:421) during data collection and analysis. An independent coder, with known academic expertise, was used. The researcher remained faithful to the academic and ethical requirements of conducting a research study.

## Results

### Step one: Concept analysis

During the concept analysis, the PNs’ experience of aggressive incidents was explored in two phases: the identification and definition of the central concept and the classification of the concepts (Chinn & Kramer 2011:163).

### Phase one: Concept identification

The concepts were derived from the researcher’s Masters’ dissertation on the lived experience of aggression and violence by nurses in a Gauteng psychiatric institution (Bimenyimana 2008:36–56). The process consisted of analysing the results and, with the available literature, finding the concept that represents the challenge that the PNs are faced with in dealing with aggression.

The central concept was identified as the ‘facilitation of the effective self-management’ of aggression.

### Phase two: Definition and classification of concepts

The ‘facilitation of effective self-management of aggression’ was defined as follows:

a dynamic interactive process through which the advanced PNP guides and provides assistance to the PNs in order to make it easier for them to take actions for the needed change. PNs are facilitated to utilise operative means and take responsibility aiming at producing the intended result, that is, the self-management of aggression. Through the process, the PNs are able to regulate their emotions and to take control of the workplace environment in effectively self-managing aggression from patients.

The concepts were classified as:

The agent is the person that makes the process of the facilitation of effective self-management of aggression to happen. In this research study, the agent is the advanced PNP who assists the PNs to find an effective way of dealing with aggression. The management also plays a role in the facilitation of effective self-management of aggression by allowing the researcher to use the facility and by providing the necessary resources, human and material, for the implementation of the model to take place.

The recipient is the person or persons who is/are the primary beneficiaries of the facilitation of effective self-management of aggression. In this study, the recipient is the PN who works in this specific psychiatric institution and participates in the search for a constructive way of managing aggression.

The context is the situation or environment in which the facilitation for effective self-management of aggression takes place. In the study, the context is a public psychiatric hospital. This psychiatric hospital is a provincial and academic hospital where psychiatric nursing students and medical students further their training in psychiatry. The main criterion for patients to be admitted is for them to be unmanageable in the referring hospitals and clinics.

The dynamics in this model consist of PNs experiencing aggression by patients. This aggression is expressed verbally, physically and emotionally and has negative personal and professional impact on them. PNs experience negative feelings of fear, anger, frustration, despair, hopelessness and helplessness prompting them to use ineffective coping mechanisms such as substance abuse, absenteeism, retaliation, a development of an ‘I don’t care attitude’ or apathy towards the work and towards what is happening around them (Bimenyimana *et al*. 2009:8).

The process for the facilitation by the researcher of effective self-management of aggression by PNs consisted of three phases: the relationship phase, the working phase and the termination phase. The process moves the PNs from the feeling of inadequacy and powerlessness to the feeling of self-confidence and taking control of the workplace challenges.

The terminus is the effective self-management of aggression by PNs.

### Step two: Relationship statements

The relationship statements were formulated as follows:

A PN who is experiencing aggression by patients and is unable to manage it effectively needs assistance and guidance in order to move from the position of victimhood to the position of taking control of the workplace aggression through a dynamic interactive process.

The advanced PNP provides the needed assistance and guidance to the PNs. This makes it easier for them to take actions for change in their workplace environment. PNs need to trust the facilitator and to believe in the process by taking responsibility for their actions or inactions in the workplace aggression.

The PN will succeed in dealing with aggressive incidents effectively by unleashing the power within and being able to regulate their emotions so that they can use their skills and knowledge in a sound manner. In embracing and implementing the alternative means, the PN would be empowered and grow both personally and professionally. They are able to take control not only of their lives and the workplace environment but also of every decision they make and how they interact with fellow professionals.

### Step three: Description of the model

#### Structure of the model

The structure of the model is discussed following the sub-headings: purpose of the model, assumptions of the model, relationship statements and the process description. The theoretical definitions of the central concept and the relationship statements have been described previously.

#### Purpose of the model

The purpose of the model was to provide a frame of reference for the advanced PN practitioner for the facilitation of effective self-management of aggression experienced by PNs working in a psychiatric institution.

#### Assumptions of the model

The assumptions of this model are embedded in the Theory for Health Promotion in Nursing (University of Johannesburg 2012:4–14). Assumptions were also inspired by another source: the Cognitive Behaviour theory (Blenkiron 2010:1–276). These assumptions are set out below.

PNs and the advanced PNP are seen wholistically; embody dimensions of body, mind and spirit; and function in an integrated manner with the environment (University of Johannesburg 2012:4).

The environment includes an internal and external environment. The internal environment consists of dimensions of body, mind and spirit. The external environment consists of physical, social and spiritual dimensions (University of Johannesburg 2012:5).

Psychiatric nursing is an interactive process for the facilitation and the promotion of mental health (University of Johannesburg 2012:5). This process is not a linear one. It requires patience, commitment, material and spiritual resources, and a clear vision in a safer workplace environment.

PNs mobilise resources by identifying and bridging the obstacles in the promotion of health (University of Johannesburg 2012:7). The identification of obstacles entails assessing the environment (both internal and external) for what it really is and strategising on how to solve the identified problems in it with the available resources. The bridging of obstacles comprises self-assessment for the strengths and weaknesses, the resources (human and materiel) required for an effective and lasting solution (University of Johannesburg 2012:7). The resources in the PNs environment are centred on the use of the self as the main actor or the scene of effective self-management of aggression.

Mental health is a dynamic interactive process in the PNs environment (University of Johannesburg 2012:5). For the PNs to feel at ease while caring for the people with mental illnesses, they must perceive the environment as safe and welcoming allowing them to maximise their potentials in fulfilling their core of duty in caring for the mentally ill patients entrusted to them. The role of the advanced PNP is to lead the way on the road which the PNs have less travelled but need to take for growth and development. PNs are allowed to work on and modify their perception about the environment in order to modify it. Blenkiron (2010:1) argues that the problem is not so much the events in one’s life, but the way in which the person interprets and acts upon them.

The facilitation of effective self-management of aggression experienced by PNs promotes their mental health when they manage to take control of the workplace environment. This facilitation requires knowledge, skills, goodwill, commitment and time from both the PNs and the advanced PNP on one side and the availability of resources and support from the nursing management on the other side.

The advanced PNP is a sensitive therapeutic professional (University of Johannesburg 2012:4) who demonstrates knowledge, skills, attitude and values in facilitating the effective self-management of aggression experienced by PNs from the patients. To achieve this, the advanced PNP must be flexible and understanding, bearing in mind that some PNs may be resistant or even hostile to change if they have come to believe that no change is possible or that the process for change may be painful compared to the situation they are in at present (McCormack & McCance 2006:476).

The workplace environment has a positive or negative impact on PNs depending on how they perceive and interact with it. Working positively on how PNs perceive workplace aggression and instilling in them hope will have a positive effect on how they self-manage this aggression effectively and will promote the mental health of the PNs and improve the quality of care rendered to the patients. Clarke (2009:843) believes that hope is the foundation of recovery from mental health problems.

#### Process description

**FIGURE 1:**
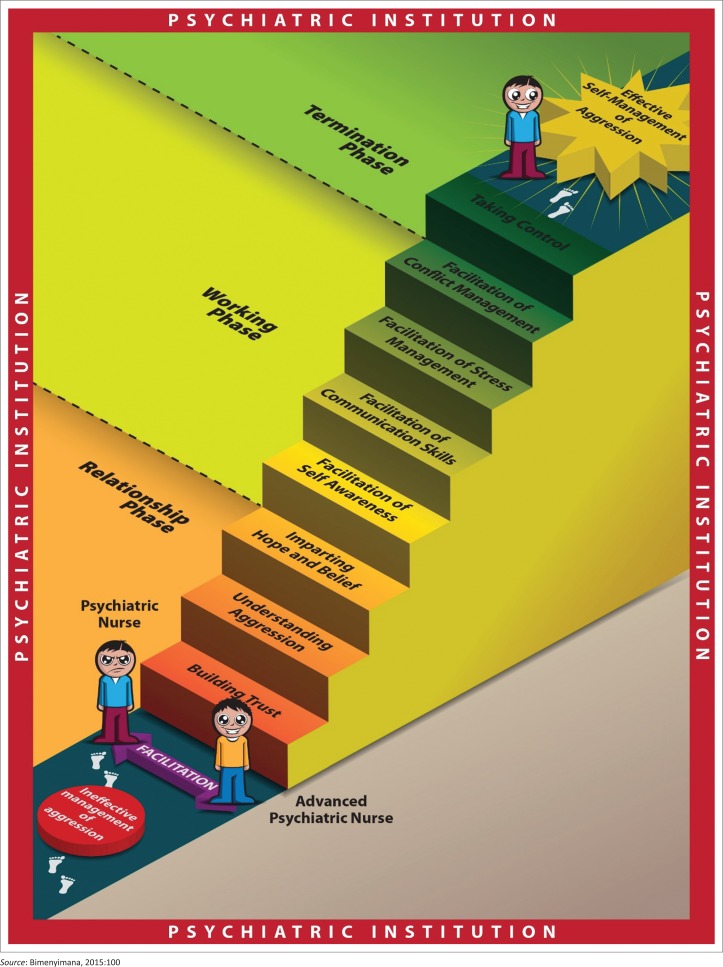
A model to facilitate effective self-management of aggression.

As displayed in Figure 1, the model structure is framed in red as an indication of potential danger the workplace environment of the PN is embedded in. It consists of three phases that are composed of steps. Each step is a building stone towards the model implementation.

The relationship phase enables both the researcher and the PNs to establish a working relation for a common purpose. It consists of three steps: (1) building trust; (2) understanding aggression; and (3) imparting hope and belief. The building of trust allows the PNs to lay down their fears and exchange information (Johnson 2003:99), coming to an understanding why the assistance is needed and how it will be achieved so that the proposed ideas can inspire confidence (Corey, Corey & Corey 2010:140). The first task in implementing any kind of change in health sector is to understand the logic of things as they are presently (Petersen *et al*. 2010:49). Hope is defined as the belief that change is possible (Corey *et al*. 2010:252). Truly speaking, hope cannot be instilled in someone. However, when suffering alone, individuals may not realise that their feelings and experiences are relatively common ones (Forsyth 2014:555–556). In sharing experiences, PNs may realise that problems have solutions and that they have the ability to improve (Robbins & Hunsaker 2012:156).

In the working phase, the identified challenges are put to the test by implementing the strategised alternatives. Corey *et al*. (2010:228) argue that the working phase is characterised by the commitment of members to explore significant challenges. The working phase consists of four steps that represent the four areas where challenges were mostly coming from. These areas are the facilitation of self-awareness; the facilitation of communication skills; the facilitation of stress management; and the facilitation of conflict management.

Sturm *et al*. (2014:659) argue that for self-awareness to successfully develop, individuals must have not only an understanding of themselves but also an understanding of and appreciation for others’ perceptions of them. The process of self-awareness entails the ability to imagine a future that is better than the past, evaluate alternatives, identify problems and a yearning to progress towards an ideal (Ashley & Reiter-Palmon 2012:1). Self-awareness also entails the change of mindset, belief, perception and attitude because how people feel about themselves has a profound effect on day-to-day functioning (Gitterman & Shulman 2005:18).

Communication plays a vital role in the management of aggression enabling both the PN and the patient to interpret both verbal and non-verbal messages correctly. Hargie and Dickson (2004:43) believe that often non-verbal communication proves decisive in conveying information and making judgements about others.

Job stress is defined as a situation wherein job-related factors interact with a worker to change his or her psychological or physiological condition such that the person is forced to deviate from normal functioning (Weinberg, Sutherland & Cooper 2010:56). Deviating from normal functioning may prove to be counter-productive in the PNs’ attempts to manage patient’s aggression. In managing aggressive incidents, one of these three ways is suggested: identifying and eliminating or minimising stressful situations, teaching the individual to cope with stress or helping those individuals who have become victims of exposure to stress (Weinberg *et al*. 2010:156). The skills that are needed in stress management are becoming aware of negative symptoms, determining the sources of stress and doing something constructive to cope with stress (Hunsaker 2005:130).

De Dreu and Gelfland (2008:6) define conflict as a process that begins when an individual or group perceives differences and opposition between itself and another individual or group about interests and resources, beliefs, values or practices that matter to them. Although conflict is an inevitable part of life and that it is prevalent among registered nurses working in the healthcare environment (Whitworth 2008:921), in conflict situations, individuals tend to develop an inflated view of their own cooperativeness and their counterpart’s hostility (De Dreu & Gelfand 2008:16). Therefore, avoiding unnecessary conflict, reducing the effect of destructive conflict, finding a solution and using any effective methods to control the direction of the conflict may help in resolving conflicts (Zhang *et al*. 2014:370).

During the termination phase, the researcher and PNs evaluate whether the intended results have been achieved. The PNs strive now to keep the gained experience while the researcher progressively withdraws from the facilitation process. Maintenance can be understood as a time of perpetual adjustment so that one prevents a falling back into older habits, but rather integrates the new self and the new behaviour into one’s daily living (Cormier, Nurius & Osborn 2013:280–281).

#### Model evaluation

The evaluation of the model was based on the criteria of clarity, simplicity, generality, accessibility and importance of the model (Chinn & Kramer 2011:196–204) as discussed below.

*Clarity*: Chinn and Kramer (2011:198) argue that in determining how clear a theory is, one should consider the following: semantic clarity, semantic consistency, structural clarity and structural consistency, that is, understanding the intended theoretical meaning of the concepts and the intended connections between concepts within the theory and the whole of the theory. The concepts were found to be meaningful, helpful and consistent. The concepts also provided a structural map that was comprehensible that was supported by logic arguments. However, some suggestions were made with regard to the order of the steps in the structure and the use of different colours. These suggestions were taken into consideration and modifications were made as suggested until all parties were satisfied.

*Simplicity*: While evaluating the model for its simplicity, the focus was on the number of elements within each descriptive category, particularly the concepts and their interrelationships (Chinn & Kramer 2011:201). The model was found to be simple.

*Generality*: The generality of a model refers to its breadth of scope and purpose (Chinn & Kramer 2011:202). The model was found to be general and that it could be applied to the PNs experiencing patients’ aggression or to all nurses working with patients.

*Accessibility*: Addresses the extent to which empirical indicators for the concepts can be identified and to what extent the purposes of the model can be attained (Chinn & Kramer 2011:203). The concepts of model were found to be accessible, *grounded and applicable to mental health nursing*.

*Importance of the model*: In nursing, the importance of the theory is closely tied to the idea of its clinical significance or practical value (Chinn & Kramer 2011:204). The evaluators stated that the model had a potential to influence nursing actions, that it could be an avenue to address a really serious challenge in interacting with challenged patients and that the model could guide nursing practice for nurses to have structured debriefing sessions to prevent secondary trauma, or as a frame of reference.

## Limitations

The model’s implementation and the evaluation of the implementation have not been published yet.

## Recommendations

The recommendations were made to the nursing practice, nursing education and nursing research. The model for the facilitation of effective self-management of aggression can enable PNs to utilise constructively the human resource and material at their disposal; it can be included in the induction programme for the newly appointed PNs. The practical relevance of the model could assist students during their practical training in the institution. It is recommended that this model be used in other areas such as private settings or other institutions where PNs are faced with the same problem of ineffective self-management of aggression. It is also recommended that the model application be researched further with different contexts and different methods.

### Original contribution of the study

The developed, described and evaluated model will be a significant contribution to the body of knowledge of psychiatric nursing and mental health. In developing a model to facilitate effective management of aggression experienced by PNs working in a psychiatric institution, the researcher will be contributing not only to the mental health of the PNs, but also to the improvement of the services rendered to the patients, the productivity of the institution and the well-being of the community and the nation at large. This research study will also inform policy-makers regarding the facilitation of mental health of mental health practitioners.

## Conclusion

The purpose of the research was to develop, describe and evaluate a model that can be used as a framework of reference for the advanced PNP to assist PNs in the effective management of aggression from the patients. The researchers shared here the model process and evaluation of the experts in the model development. The step by step of how the model was developed was also described as well as the ethical consideration and the measures taken to ensure trustworthiness. The limitations and recommendations were also discussed. It is hoped that, in the future article, the researchers will share how the model was implemented by PNs and what their experience was.
